# Prognostic Significance of Cyclin D1 Expression in Colorectal Cancer: A Meta-Analysis of Observational Studies

**DOI:** 10.1371/journal.pone.0094508

**Published:** 2014-04-11

**Authors:** Yang Li, Jun Wei, Chuanhui Xu, Zhongxin Zhao, Tiangeng You

**Affiliations:** 1 Department of General surgery, East Hospital, Tongji University School of Medicine, Shanghai, China; 2 Department of Radiology, Qingpu Branch of Zhongshan Hospital, Fudan University, Shanghai, China; National Cancer Center, Japan

## Abstract

**Objective:**

Cyclin D1 plays a vital role in cancer cell cycle progression and is overexpressed in many human cancers, including colorectal cancer (CRC). However, the prognostic value of cyclin D1 overexpression in colorectal cancer is conflicting and heterogeneous. We conducted a meta-analysis to more precisely evaluate its prognostic significance.

**Methods:**

A comprehensive literature search for relevant studies published up to January 2014 was performed using PubMed, EMBASE, and ISI Web of Science. The pooled hazard ratio (HR) with 95% confidence intervals (CI) was used to estimate the effects.

**Results:**

22 studies with 4150 CRC patients were selected to evaluate the association between cyclin D1 and overall survival (OS), disease-free survival (DFS) and clinicopathological parameters. In a random-effects model, the results showed that cyclin D1 overexpression in CRC was significantly associated with both poor OS (HR = 0.73, 95% CI: 0.63–0.85, *P*<0.001) and DFS (HR = 0.60, 95% CI: 0.44–0.82, *P* = 0.001). Additionally, cyclin D1 overexpression was significantly associated with more relative older patients (≥60 years) (OR 0.62, 95% CI 0.44–0.89, *P* = 0.009), T3,4 tumor invasion (OR 0.70, 95% CI 0.57–0.85, *P*<0.001), N positive (OR 0.75, 95% CI 0.60–0.95, *P* = 0.016) and distant metastasis (OR 0.60, 95% CI 0.36–0.99, *P* = 0.047) of CRC.

**Conclusion:**

The meta-analysis results indicated that cyclin D1 is an unfavorable prognostic factor for CRC. Cyclin D1 overexpression might be associated with poor clinical outcome and some clinicopathological factors such as age, T category, N category and distant metastasis in CRC patients.

## Introduction

Colorectal cancer (CRC) is the third most frequent malignancy worldwide and the fourth most frequent cause of death from cancer in the world [1]. The incidence of CRC in China is lower than that in western countries, but has increased in recent years, particularly in more developed areas [2]. Despite the development of combined therapeutic modalities and the prolonged overall survival (OS) and disease-free survival (DFS) of CRC patients, CRC remains the second leading cause of overall cancer deaths [3]. It is valuable to identify molecular predictive markers for the prognosis, which would be helpful in the selection of therapeutic strategies and further improve patients' survival for CRC.

Much attention has been focused on the involvement of cyclin D1 in tumor development and progression [4]. Cyclin D1 has been considered to be an oncogene which could regulate progression from the G1 phase of the cell cycle to the S phase [5]. As known to us, the ability of cyclin D1 to drive the cell cycle forward can be blocked by cyclin D1-dependent kinase (CDK) inhibitors, such as p27 and p21. As key regulators of the G1 progression step within the cell cycle, cyclin D1 have been suspected to play a pivotal role in the process of carcinogenesis and cancer progression [6]. Cyclin D1 expression is known to be upregulated in a variety of tumor types and occurs in one-third or more of colorectal cancers [7]–[17]. Many studies have evaluated whether cyclin D1 overexpression may be a prognostic factor for survival in patients with CRC. However, the results of the studies are inconclusive and no consensus has been reached. Bahnassy *et al*. [10] and Maeda *et al*. [9] reported that cyclin D1 overexpression has been associated with poor prognosis, while Holland *et al*. [11] and Ogino *et al*. [18] draw a conclusion that the high level of cyclin D1 indicate good prognosis. A few studies have shown no prognostic value of cyclin D1 overexpression [8], [12], [19]. When it comes to the associations between cyclin D1 expression and clinicopathological parameters, the studies were also heterogeneous [10], [15]–[17], [20]–[25], [46]. It is necessary to establish whether cyclin D1 overexpression is a prognostic marker in CRC.

In this meta-analysis, we collected and combined all eligible published articles about the relation between cyclin D1 and survival in CRC. The aim of our study was to test the hypothesis that cyclin D1 overexpression would predict the clinical outcomes of patients with CRC. Additionally, the relation between cyclin D1 expression and clinicopathological parameters were examined.

## Materials and Methods

### Search Strategy

We searched PubMed, EMBASE and ISI Web of Science to identify studies assessing the cyclin D1 as prognostic factor in patients with CRC. The upper data limit of January 2014 was applied, with no lower date limit. The search strategy performed in PubMed combined the following terms: (colorectal OR colon OR rectum OR colorectum OR large bowel OR gut) AND (cancer* OR carcinoma* OR neoplasm* OR tumor* OR polyp*) AND (cyclin d1 protein OR CCND 1 OR cyclin D1 OR cyclin-D1) AND (prognosi*) (Table S1). The search was limited to human studies. The similar search strategy was used in other databases. The language of all publications was limited to English only. The title and abstract of each study identified in the search was scanned to exclude any clearly irrelevant ones. The reference lists of each identified study were also reviewed to identify the additional studies containing information on the topic of interest.

### Study Selection

Criteria for eligibility of a study included in this meta-analysis were: (1) to assess cyclin D1 expression in the primary colorectal cancer tissues using immonohistochemistry (IHC) (not in metastatic tissue or mucosa adjacent to the tumor); (2) the endpoint investigated was OS, DFS; (3) the study reported a hazard ratio (HR) estimates with their 95% confidence intervals (CI) or the data sufficient allowing for estimation of the HR and 95% CI from survival analysis; (4) to be published as a full paper in the English language; (5) when the same author reported results from the same patient population, the most recent report or the most complete one was included. Articles that could not be identified based on title and abstract alone were retrieved for full-text review. To determine the issue of multiple publications from the same data sets, we checked all author names, institutions involved, and the time period of patient recruitment of the articles.

### Data Extraction

Two authors (Li Y. and Wei J.) independently reviewed each eligible study and extracted data with a standardized protocol (Text S1) and predefined data collection form (Excel sheet). Disagreements were resolved with third author (You T. G.) through discussion. Information was carefully retrieved from the full publications, including the following items: the first author, year of publication, study location, number of participants, staining patterns of cyclin D1, the choice of cutoff scores for the definition of positive staining or staining intensity, antibody used, antibody working concentration, duration of follow-up, T category, N category, distant metastasis, histology, and prognostic outcomes of interest (DFS and/or OS). As the cutoff value for cyclin D1-high group varied with different studies, we defined cyclin D1-high expression values according to the original articles. Staging of CRC was based on the UICC classification revised in 2009 [26]. Tumor differentiation was graded by a pathologist according to the World Health Organization (WHO) classification system. The primary authors were contacted to provide additional information when necessary.

### Assessment of Study Quality

Two authors (Li Y. and Xu C. H.) independently assessed the quality of all studies on the basis of a 9-scores system of the Newcastle-Ottawa Scale (NOS) [27]. Discrepancies in the score were resolved through discussion between the authors. Each study included in the meta-analysis was judged on three broad perspectives: (1) the selection of the groups of study (four items, one score each), (2) the comparability (one item, up to two scores) and (3) the ascertainment of either the exposure or outcome of interest (three items, one score each). A score presents a high quality choice of individual study. In this 9-scores system, studies scored equal or greater than 7 were considered as high quality.

### Statistical Methods

Included studies were divided into two groups for analysis: OS and DFS. For the quantitative aggregation of the survival results, we measured the impact of cyclin D1 overexpression on survival by HR between the two survival distributions. HR and 95% confidence intervals (CI) were used to combine as the effective value. If these statistical variables were not given explicitly in an article, they were estimated from available data using methods reported by Tierney and colleagues [28]. Kaplan-Meier curves were read using Engauge Digitizer version 4.1 (http://digitizer.sourceforge.net/), and then the survival data read from Kaplan-Meier curves were entered in the spreadsheet based on Tierney [28]. For the pooled analysis of the relation between cyclin D1 overexpression and clinicopathological parameters (age, tumor size, T category, N category, distant metastasis, histological grade), OR and their 95% CI were combined to give the effective value. The individual HR estimates were pooled into a summary HR by using DerSimonian and Laird random-effects methods reported by Yusuf *et al*. [29]. The random-effects model, which not only weights each study by its inverse variance but also includes the within- and between-studies variances and thus is usually more conservative, was chosen. Statistical heterogeneity assessment between studies was performed by using a Chi-square heterogeneity statistic based *Q* test. Given the low test power, the significance level was defined as *P*<0.10. The effect of heterogeneity was also quantified using the inconsistency index (*I* ^2^). The *I* ^2^ statistic is defined as the percentage of total variance across studies attributable to heterogeneity rather than the chance [*I* ^2^ = (*Q* – df)/*Q*×100%]. As a guide, *I* ^2^ values of <25% may be considered “low”, values of 25–50% may be considered “moderate” and values of >50% may be considered “high” [30]. For OS, subgroup analyses were performed by treatment (single surgery and surgery as well as chemoradiation), geographic settings (Asian and non-Asian CRC patients), samples (whole tissue sections and tissue microarray), staining patterns (nuclear, nuclear & cytoplasmic and single cytoplasmic staining), study quality (≥7 and <7), study design (cohort studies and case-control studies). For DFS, subgroup analyses were performed by treatment, geographic settings, staining patterns, study design and study quality.

We also carried out sensitivity analysis to evaluate the influence of a single study on the overall effect estimate by excluding one study at a time. The potential for publication bias was assessed by using the Begg rank correlation method and the Egger weighted regression method (*P*<0.05 was considered representatively of statistically significant publication bias). The meta-analysis including metan, metainf, and metabias command was performed by Stata 11.0 software (Stata Corp, College Station, TX, USA). A *P* value less than 0.05 was considered to be statistically significant except where otherwise specified.

## Results

### Search Results and Study Characteristics

**Figure 1:**
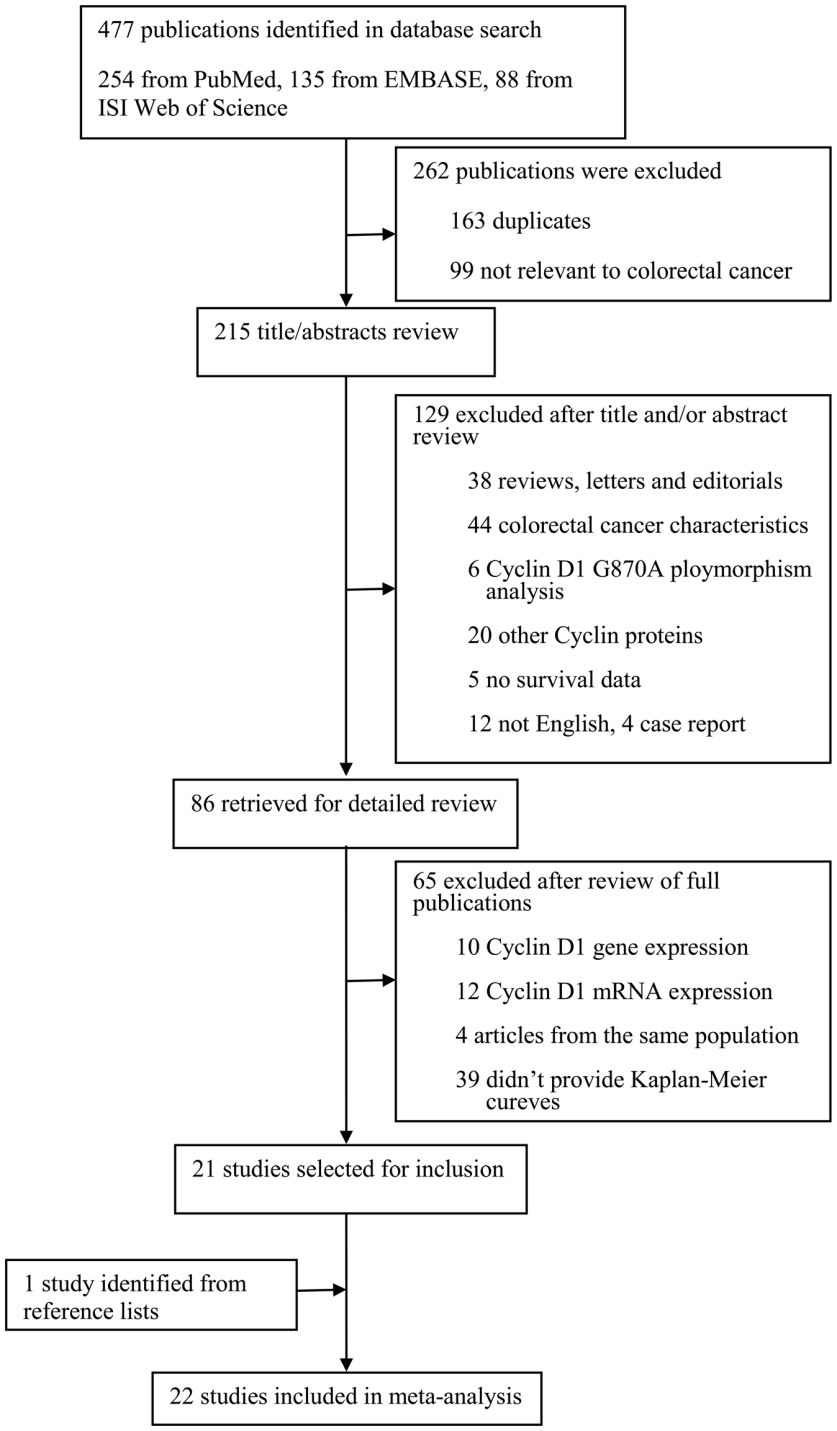
Flow diagram of screened, excluded, and analyzed publications.

A total of 477 potentially relevant publications were retrieved after the initial database searches, and 22 observational studies met the predefined inclusion criteria comprising 4150 patients for final analysis [8]–[10], [13]–[25], [31]–[35], [47]. On the basis of full text review, we identified 21 studies [9]–[10], [13]–[25], [31]–[35], [47]. One study was identified from reference lists [8]. A flow diagram of the study selection process is presented in Figure 1. The major characteristics of the 22 eligible studies were reported in Table 1. The sample size of the included studies ranged from 39 to 602 patients (median sample size, 188.6 patients) and follow-up period vary from 30 to 107 months. The studies were conducted in 14 countries (China, Egypt, Finland, Germany, Greece, India, Japan, Korea, Netherlands, Norway, Poland, Sweden, United Kingdom and United States) and published between 1996 and 2013. Among the 22 studies, 8 studies (1395 patients, 33.6%) were performed in Asian populations [9], [13], [20], [22], [24], [31], [34], [47], and the remaining studies (2755 patients, 66.4%) followed non-Asian patients [8], [10], [14]–[19], [21], [23], [25], [32]–[33], [34].

**Table 1:** Characteristics of studies included in the meta-analysis.

First author of study [ref.]	Year	Country	Design	Number of patients	Duration of follow-up	Method to determine "high" Cyclin D1 cut-off level (high/low)	Scores of study quality	Adjusted confounders	Outcome	HR estimation
Bahnassy[10]	2004	Egypt	Case control	60	*	Staining index ≥6.1 (41/19)	7	Age, gender, the depth of invasion, stage, lymph node metastasis, cyclin A expression	OS, DFS	Data extrapolated
Wang[31]	1996	Japan	Case control	39	*	Tumor/mucosa ratio >1.3 (4/35)	6	_	NA	_
Balcerczak[25]	2005	Poland	Case control	111	*	>25% (69/42)	7	Age, gender, histological type, stage	OS, DFS	Reported in text
Tsai[22]	2013	Taiwan, China	Prospective cohort	100	Median 30.5 months	Score ≥2 (49/51)	7	Vascular invasion, stage, VEGF expression, postoperative CEA	OS, DFS	Data extrapolated
Mckay[15]	2002	UK	Prospective cohort	249	Median 35 months	>5% (137/112)	8	Age, stage	OS	Data extrapolated
Hilska[17]	2005	Finland	Prospective cohort	363	NA	>1% (99/264)	7	T stage, modified Dukes stage, histological differentiation, urgency of operation, Ki-67 labeling, CEA expression	OS	Reported in text
Theocharis[23]	2007	Greece	Prospective cohort	86	Median 43 months	>5% (56/30)	7	Age, gender, tumor location, histological stage and grade, lymph node and liver metastasis, venous invasion	OS	Reported in text
Von Wangenheim[21]	2007	Germany	Prospective cohort	200	At least 5 years	>5% (76/124)	8	Age	OS, DFS	Data extrapolated
Fang[34]	2009	China	Prospective cohort	532	Median 52 months	>10% (380/152)	6	Age, MMP7, Survivin, TROP2, pathology grade, bowel wall invasion, lymph node metastasis, during-operative chemotherapy	OS	Data extrapolated
Mao[24]	2011	China	Prospective cohort	169	3 to 107 months	>5% (95/74)	7	T stage, lymph node metastasis, distant metastasis, TNM stage, P-Stat5 expression	OS	Data extrapolated
Saridaki[32]	2010	Greece	Prospective cohort	144	NA	≥20% (26/118)	6	BRAF status, stage, metastasectomy, number of treatment lines	OS, DFS	Data extrapolated
Bhatavdekar[13]	2001	India	Prospective cohort	98	5 years	Score ≥1 (30/68)	7	Dukes stage, CD44, CK-19, PRL	OS, DFS	Reported in text
Palmqvist[8]	1998	Sweden	Prospective cohort	90	Median 42 months	>50% (11/79)	7	Dukes stage, pRb expression,	OS	Data extrapolated
Belt[35]	2012	Netherlands	Prospective cohort	379	NA	Score ≥8 (168/211)	8	Age, gender, stage, tumor location, lymph node yield, MSI status, venous invasion, chemotherapy treatment	OS, DFS	Data extrapolated
Pasz-Walczak[14]	2001	Poland	Prospective cohort	122	Median 44.5 months	>50% (68/54)	7	Lymph node invasion, stage, hepatic metastasis, p21	OS	Reported in text
Moore[33]	2004	USA	Prospective cohort	40	Median 69 months	>10% (6/34)	8	Lymph node invasion, the depth of tumor invasion, stage, p27, p53	OS, DFS	Data extrapolated
Bondi[16]	2005	Norway	Prospective cohort	219	5 years	>5% (24/195)	6	Age	OS, DFS	Data extrapolated
Maeda[9]	1997	Japan	Prospective cohort	101	5 years	>50% (14/87)	7	Histological differentiation, stage, lymph node metastasis, the depth of invasion, lymphatic invasion, venous invasion, p53	OS, DFS	Reported in text
Ogino[18]	2009	USA	Prospective cohort	602	Every 2 years	>50% (330/272)	8	Age, gender, year of diagnosis, BMI, family history of CRC in any first-degree relative, tumor location, stage, grade, status of MSI, CIMP, LINE-1, KRAS, BRAF, p53, p21, p27, COX-2, FASN	OS	Reported in text
Wang[20]	2013	China	Case control	139	*	>5% (83/56)	6	Differentiation, TNM stage, YAP expression	OS	Data extrapolated
Jang[47]	2012	Korea	Prospective cohort	217	NA	≥30% (129/88)	7	Age, gender, tumor location, tumor size, differentiation, lymphovascular invasion, stage, preoperative CEA, CA19-9 level, β-catenin expression	OS	Reported in text
Lyall[19]	2012	UK	Prospective cohort	90	60 to 100 months	>5% (46/44)	8	Age, gender, cluster group, tumor location, status of apical node, stage, nodal status, tumor differentiation	OS	Data extrapolated

*these studies looked back at medical records and did not report the time of follow-up.

Abbreviations: BMI: body mass index; CA19-9: carbohydrate antigen 19-9; CEA: carcinoembryonic antigen; CIMP: the CpG island methylator phenotype; CK-19: cytokeratin-19; COX-2: cyclooxygenase-2; CRC: colorectal cancer; DFS, disease-free survival; FASN: fatty acid synthase; HR: hazard ratio; LINE-1: long interspersed nucleotide element-1; MMP-7: matrix metalloproteinase-7; MSI: microsatellite instability; NA: not available; OS: overall survival; pRb: retinoblastoma protein; PRL: prolactin; P-Stat5: phosphorylated signal transducer and activator of transcription-5; VEGF: vascular endothelial growth factor; YAP: Yes-associated protein.

Of the 22 studies, 21 studies reported the prognostic value of cyclin D1 expression for OS in patients with colorectal cancer [8]–[10], [13]–[25], [32]–[35], [47]. Regarding treatment, colorectal cancer can be treated with either surgery, radiotherapy, chemotherapy or a combination of these treatments. The study could be classified two subgroups according to whether the patients received chemoradiation in addition to surgical operation. In subgroup analysis, 16 studies were treated by single surgery [8]–[10], [14]–[25], [35], while 5 studies were treated with surgery as well as chemoradiation treatment [13], [32]–[34], [47]. 7 studies (1346 patients, 32.7%) were performed in Asian populations [9], [13], [20], [22], [24], [34], [47], and the remaining 14 studies (2765 patients, 67.3%) followed non-Asian patients [8], [10], [14]–[19], [21], [23], [25], [32]–[33], [34]. 17 studies used whole tissue sections to detect cyclin D1 antigen [8]–[10], [13]–[17], [19]–[25], [32]–[33], while 4 studies used tissue microarray [18], [34]–[35], [47]. With respect to the staining patterns, 13 studies detected the cyclin D1 with nuclear cyclin D1 staining [9], [14], [16], [18]–[20], [22], [23], [25], [32]–[33], [35], [47], 7 studies with combined nuclear and cytoplasmic cyclin D1 staining [8], [10], [15], [17], [21], [24], [34], and 1 study with cytoplasmic only [13]. 4 studies were classified into low quality group [16], [20], [32], [34], and 17 studies were high quality group [8]–[10], [13]–[15], [17]–[19], [21]–[25], [33], [35], [47]. 3 studies were case-control studies [10], [20], [25] and 18 were prospective cohort studies [8]–[9], [13]–[19], [21]–[24], [32]–[35], [47]. DFS was obtained in ten studies [9]–[10], [13], [16], [21]–[22], [25], [32]–[33], [35]. In subgroup analysis defined by geographic settings, 3 studies (299 patients, 20.6%) were performed in Asian populations [9], [13], [22], and 7 studies (1153 patients, 79.4%) followed non-Asian populations [10], [16], [21], [25], [32]–[33], [35]. Among these studies, seven were treated by single surgery [9]–[10], [16], [21]–[22], [25], [35], while three were undergone surgery and chemoradiation therapies [13], [32]–[33]. When grouped according to the staining patterns, cyclin D1 was detected by nuclear staining in 7 studies [9], [16], [22], [25], [32]–[33], [35], by nuclear and cytoplasmic staining in 2 studies [10], [21], and by single cytoplasmic staining only in 1 study [13]. With respect to the study design, two were case-control studies [10], [25] and eight were prospective cohort studies [9], [13], [16], [21]–[22], [32]–[33], [35]. According to the Newcastle-Ottawa quality assessment scale, 2 studies were classified into low quality group [16], [32], and 8 studies were high quality group [9]–[10], [13], [21]–[22], [25], [33], [35].

### Methodological Quality of the Studies

To assess the quality of the included studies, two authors (Li Y. and Xu C. H.) independently extracted data and assessed the methodological quality using the Newcastle-Ottawa quality assessment scale. The scores of the included studies ranged from 6 to 8 (with a mean of 7.05), which were shown in Table 1. The studies included in our meta-analysis had moderate or high levels of methodological quality. Table S2 summarizes the quality scores of each item of cohort studies and case-control studies.

### Quantitative synthesis

#### Impact of cyclin D1 expression on OS of CRC

**Figure 2:**
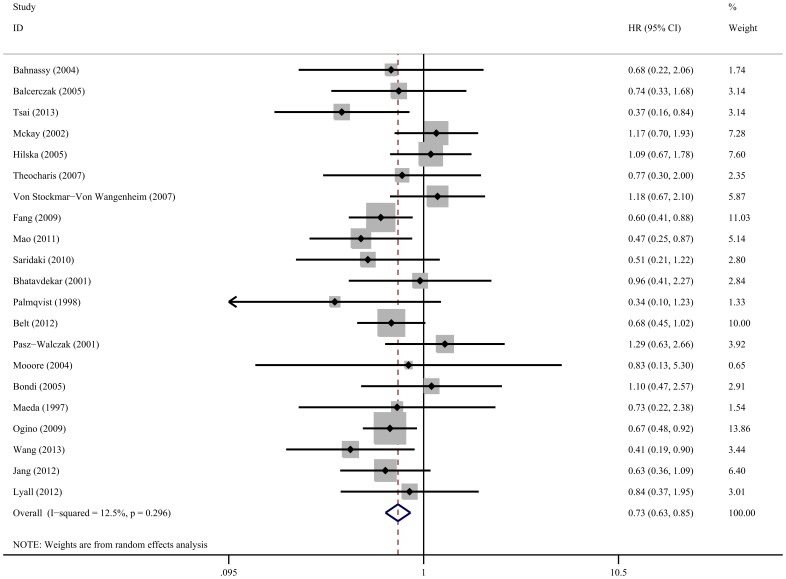
Forest plot of the hazard ratio (HR) for the association of cyclin D1 expression with overall survival (OS). Horizontal lines represent 95% CI. Each box represents the HR point estimate, and its area is proportional to the weight of the study. The diamond (and broken line) represents the overall summary estimate, with CI represented by its width. The unbroken vertical line indicates the null value (HR = 1).

Meta analysis of 21 studies on the prognostic value of cyclin D1 expression showed that high cyclin D1 levels were associated with poor OS (HR obtained from random-effects model: 0.73, 95% CI: 0.63–0.85, *P*<0.001), without significant heterogeneity between studies (*I* ^2^ = 12.5%, *P* = 0.296) (Figure 2). Subgroup analysis indicated that when grouped according to geographic settings of individual studies, the pooled HR of Asian studies and non-Asian studies were 0.56 (95% CI: 0.45–0.72, *P*<0.001, without significant heterogeneity) and 0.83 (95% CI: 0.70–0.98, *P* = 0.026, and without significant heterogeneity), respectively, indicating that cyclin D1 is an indicator of poor OS both in Asian patients and non-Asian patients (Table 2). Cyclin D1 overexpression was related markedly with poor OS in colorectal cancer patients treated by single surgery (HR: 0.77, 95% CI: 0.63–0.93, *P* = 0.006) and surgery as well as chemoradiation (HR: 0.63, 95% CI: 0.48–0.83, *P* = 0.001), without significant heterogeneity between studies. Patients with high cyclin D1 expression based on the whole tissue sections seemed to have worse OS than those with cyciln D1 low expression group (HR: 0.79, 95% CI: 0.64–0.98, *P* = 0.032, and *I* ^2^ = 18.9%, *P* = 0.233 for heterogeneity). Cyclin D1 overexpression detected by tissue microarray was also associated with a worse OS (HR: 0.64, 95% CI: 0.53–0.79, *P*<0.001, without significant heterogeneity). When grouped according to staining patterns, cyclin D1 overexpression had significant impact on OS with nuclear staining (HR: 0.68, 95% CI: 0.57–0.81, *P*<0.001, without significant heterogeneity) but not for nuclear combining with cytoplasmic staining (HR: 0.79, 95% CI: 0.57–1.10, *P* = 0.159, *I* ^2^ = 51.2%, *P* = 0.056 for heterogeneity) or only cytoplasmic staining (HR: 0.96, 95% CI: 0.41–2.27, *P* = 0.922). Subgroup analysis by study design suggested that cyclin D1 overexpression predicted poor OS in both case-control studies (HR: 0.57, 95% CI: 0.35–0.94, *P* = 0.028, without significant heterogeneity) and prospective cohort studies (HR: 0.75, 95% CI: 0.64–0.89, *P* = 0.001, *I^2^* = 17.8%, *P* = 0.241 for heterogeneity). The study quality subgroup analysis indicated a significant relation between cyclin D1 overexpression and poor OS, which was exhibited in both low-quality studies (HR: 0.60, 95% CI: 0.44–0.81, *P* = 0.001, without significant heterogeneity) and high-quality studies (HR: 0.77, 95% CI: 0.65–0.91, *P* = 0.002, *I^2^* = 9.7%, *P* = 0.341 for heterogeneity).

**Table 2:** Results of overall and subgroup analyses for effects of cyclin D1 expression on overall and disease-free survival in colorectal cancer.

Categories	N	Patients	References	Random-effects model		Heterogeneity		
				HR (95% CI)	*P*	*Q*	*I* ^2^	*P*
Overall survival (OS)	21	4111	8–10, 13–25, 32–35, 47	0.73 (0.63–0.85)	<0.001	22.85	12.5%	0.296
								
Subgroup 1: Single surgery	16	3080	8–10, 14–25, 35	0.77 (0.63–0.93)	0.006	20.09	25.3%	0.169
								
Surgery and chemoradiation	5	946	13, 32–34, 47	0.63 (0.48–0.83)	0.001	1.30	0%	0.861
								
								
Subgroup 2: Asian	7	1362	9, 13, 20, 22, 24, 34, 47	0.56 (0.45–0.72)	<0.001	3.82	0%	0.700
								
Non-Asian	14	2749	8, 10, 14–19, 21, 23, 25, 32–34	0.83 (0.70–0.98)	0.026	12.21	0%	0.511
								
Subgroup 3: Whole tissue sections	17	2381	8–10, 13–17, 19–25, 32–33	0.79 (0.64–0.98)	0.032	19.73	18.9%	0.233
								
Tissue microarray	4	1730	18, 34–35, 47	0.64 (0.53–0.79)	<0.001	0.26	0%	0.968
								
Subgroup 4: Nuclei	13	2350	9, 14, 16, 18–20, 22, 23, 25, 32–33, 35, 47	0.68 (0.57–0.81)	<0.001	8.90	0%	0.712
								
Nuclei and cytoplasm	7	1663	8, 10, 15, 17, 21, 24, 34	0.79 (0.57–1.10)	0.159	12.30	51.2%	0.056
								
Cytoplasm	1	98	13	0.96 (0.41–2.27)	0.922	0	-	-
								
Subgroup 5: Case control studies	3	310	10, 20, 25	0.57 (0.35–0.94)	0.028	1.15	0%	0.563
Prospective cohort studies	18	3801	8– 9, 13–19, 21–24, 32–35, 47	0.75 (0.64–0.89)	0.001	20.68	17.8%	0.241
Subgroup 6: Low quality studies	4	1034	16, 20, 32, 34	0.60 (0.44–0.81)	0.001	2.98	0%	0.395
High quality studies	17	3077	8–10, 13–15, 17–19, 21–25, 33, 35, 47	0.77 (0.65–0.91)	0.002	17.72	9.7%	0.341
Disease-free survival (DFS)	10	1452	9–10, 13, 16, 21–22, 25, 32–33, 35	0.60 (0.44–0.82)	0.001	11.80	23.7%	0.225
								
Subgroup 1: Single surgery	7	1170	9–10, 16, 21–22, 25, 35	0.69 (0.51–0.92)	0.011	6.56	8.5%	0.364
								
Surgery and chemoradiation	3	282	13, 32–33	0.33 (0.17–0.63)	0.001	1.17	0%	0.556
								
								
Subgroup 2: Asian	3	299	9, 13, 22	0.41 (0.24–0.72)	0.002	0.44	0%	0.804
								
Non-Asian	7	1153	10, 16, 21, 25, 32–33, 35	0.67(0.46–0.98)	0.041	8.92	32.7%	0.178
								
Subgroup 3: Nuclei	7	1094	9, 16, 22, 25, 32–33, 35	0.54 (0.38–0.77)	0.001	7.66	21.7%	0.264
Nuclei and cytoplasm	2	260	10, 21	1.00 (0.57–1.77)	0.997	0.56	0%	0.453
Cytoplasm	1	98	13	0.46 (0.18–1.18)	0.107	0	-	-
Subgroup 4: Case control studies	2	171	10, 25	0.72 (0.37–1.40)	0.329	0.02	0%	0.885
Prospective cohort studies	8	1281	9, 13, 16, 21–22, 32–33, 35	0.57 (0.39–0.83)	0.004	11.54	39.3%	0.117
Subgroup 5: Low quality studies	2	363	16, 32	0.52 (0.12–2.24)	0.381	4.34	77.0%	0.037
High quality studies	8	1089	9–10, 13, 21–22, 25, 33, 35	0.61 (0.46–0.82)	0.001	7.46	6.1%	0.383

Abbreviations: HR: hazard ratio; 95% CI: 95% confidence interval; N: number of studies.

#### Cyclin D1 expression and DFS in CRC

**Figure 3:**
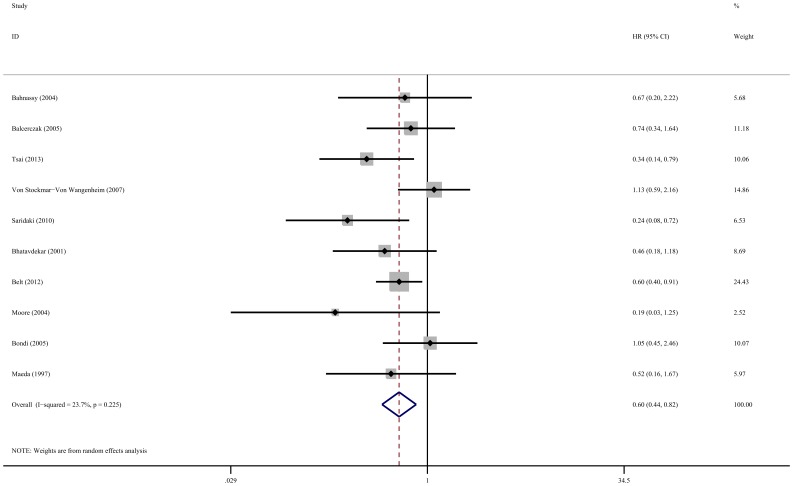
Forest plot of the hazard ratio (HR) for the association of cyclin D1 expression with disease-free survival (DFS).

Meta-analysis of 10 studies showed that high cyclin D1 expression was associated with poor DFS in colorectal cancer patients (HR: 0.60, 95% CI: 0.44–0.82, *P* = 0.001, and *I* ^2^ = 23.7%, *P* = 0.225 for heterogeneity) (Figure 3). When grouped according to geographic settings, the pooled HR of Asian studies and non-Asian studies were 0.41 (95% CI: 0.24–0.72, *P* = 0.002, without significant heterogeneity) and 0.67 (95% CI: 0.46–0.98, *P* = 0.041, and *I* ^2^ = 32.7%, *P* = 0.178 for heterogeneity), respectively (Table 2). The results showed that cyclin D1 overexpression were markedly associated with poor DFS in both Asian and non-Asian CRC patients. Restricting the analysis to studies that treated by single surgery (HR: 0.69, 95% CI: 0.51–0.92, *P* = 0.011, and *I* ^2^ = 8.5%, *P* = 0.364 for heterogeneity) and surgery as well as chemoradiation subgroups (HR: 0.33, 95% CI: 0.17–0.63, *P* = 0.001, without significant heterogeneity) also indicated the difference in DFS between cyclin D1-high and low level groups. When grouped according to staining patterns, cyclin D1 overexpression had significant impact on DFS with only nuclear staining (HR: 0.54, 95% CI: 0.38–0.77, *P* = 0.001, *I^2^* = 21.7%, *P* = 0.264 for heterogeneity) but not for nuclear and cytoplasmic staining (HR: 1.00, 95% CI: 0.57–1.77, *P* = 0.997, without significant heterogeneity) and single cytoplasmic staining (HR: 0.46, 95% CI: 0.18–1.18, *P* = 0.107). Furthermore, subgroup analysis revealed that the significant correlation between cyclin D1 overexpression and worse DFS in prospective cohort studies (HR: 0.57, 95% CI: 0.39–0.83, *P* = 0.004, *I^2^* = 39.3%, *P* = 0.117 for heterogeneity) but not in case-control studies (HR: 0.72, 95% CI: 0.37–1.40, *P* = 0.329, without significant heterogeneity). The study quality subgroup analysis shown that a significant relation between high level cyclin D1 and poor DFS in high quality studies (HR: 0.61, 95% CI: 0.46–0.82, *P* = 0.001, *I^2^* = 6.1%, *P* = 0.383 for heterogeneity) but not in low quality studies (HR: 0.52, 95% CI: 0.12–2.24, *P* = 0.381, *I^2^* = 77.0%, *P* = 0.037 for heterogeneity).

#### Cyclin D1 expression and clinicopathological parameters

The association between cyclin D1 and several clinicopathological parameters was illustrated in Figure S1, S2, S3, S4, S5, and S6. Eleven studies reported data on the correlation between cyclin D1 expression and colorectal cancer patients' age [13]–[14], [17], [20], [22], [24], [31]–[32], [34]–[35], [47]. The pooled OR was 0.62 (95% CI: 0.44–0.89, *P* = 0.009), suggesting elder patients (≥60 years) had significantly higher cyclin D1 expression than younger patients (<60 years) (Table 3). Furthermore, six studies reported data on tumor size [10], [20], [22], [24], [35], [47], sixteen studies reported data on T category [8]–[10], [14]–[15], [17]–[18], [20]–[22], [24]–[25], [31], [34]–[35], [47], fourteen studies reported data on N category [9]–[10], [14]–[15], [20]–[25], [31], [34]–[35], [47], nine studies reported data on distant metastasis [14], [20], [22]–[25], [31], [34], [47], eighteen studies reported data on histology and their relationship with cyclin D1 expression [8]–[10], [13]–[15], [17]–[18], [20]–[24], [31]–[32], [34]–[35], [47]. When the data was pooled, there were significant association between high cyclin D1 expression and the clinicopathological parameters except tumor size and histology. Specifically, the pooled OR were as follows: 0.64 (0.35–1.16, *P* = 0.139) for tumor size (<5 cm vs. ≥5 cm), 0.70 (0.57–0.85, *P*<0.001) for T category (T1,2 vs. T3,4), 0.75 (0.60–0.95, *P* = 0.016) for N category (negative vs. positive), 0.60 (0.36–0.99, *P* = 0.047) for distant metastasis (M0 vs. M1), 0.87 (0.71–1.07, *P* = 0.178) for histology (Well, moderate vs. poor).

**Table 3:** Meta-analysis of the association between cyclin D1 expression and clinicopathological features of colorectal cancer.

Categories	N	Patients	References	Random-effects model		Heterogeneity		
				OR (95% CI)	*P*	*Q*	*I* ^2^	*P*
Age in years (<60 vs. ≥60)	11	2302	13–14, 17, 20, 22, 24, 31–32, 34–35, 47	0.62 (0.44–0.89)	0.009	32.79	69.5%	<0.001
Tumor size (cm) (<5 vs. ≥5)	6	1064	10, 20, 22, 24, 35, 47	0.64 (0.35–1.16)	0.139	23.81	79.0%	<0.001
T category (T1,2 vs. T3,4)	16	3473	8–10, 14–15, 17–18, 20–22, 24–25, 31, 34–35, 47	0.70 (0.57–0.85)	<0.001	21.24	29.4%	0.129
								
N category (negative vs. positive)	14	2504	9–10, 14–15, 20–25, 31, 34–35, 47	0.75 (0.60–0.95)	0.016	20.55	36.7%	0.082
								
Distant metastasis (M0 vs. M1)	9	1376	14, 20, 22–25, 31, 34, 47	0.60 (0.36–0.99)	0.047	23.03	65.3%	0.003
								
Histology (Well,mod vs. por)	18	3688	8–10, 13–15, 17–18, 20–24, 31–32, 34–35, 47	0.87 (0.71–1.07)	0.178	19.12	11.1%	0.321

Abbreviations: OR: odds ratio; 95% CI: 95% confidence interval; mod: moderate; por: poor; N, number of studies.

### Sensitivity analyses

**Figure 4:**
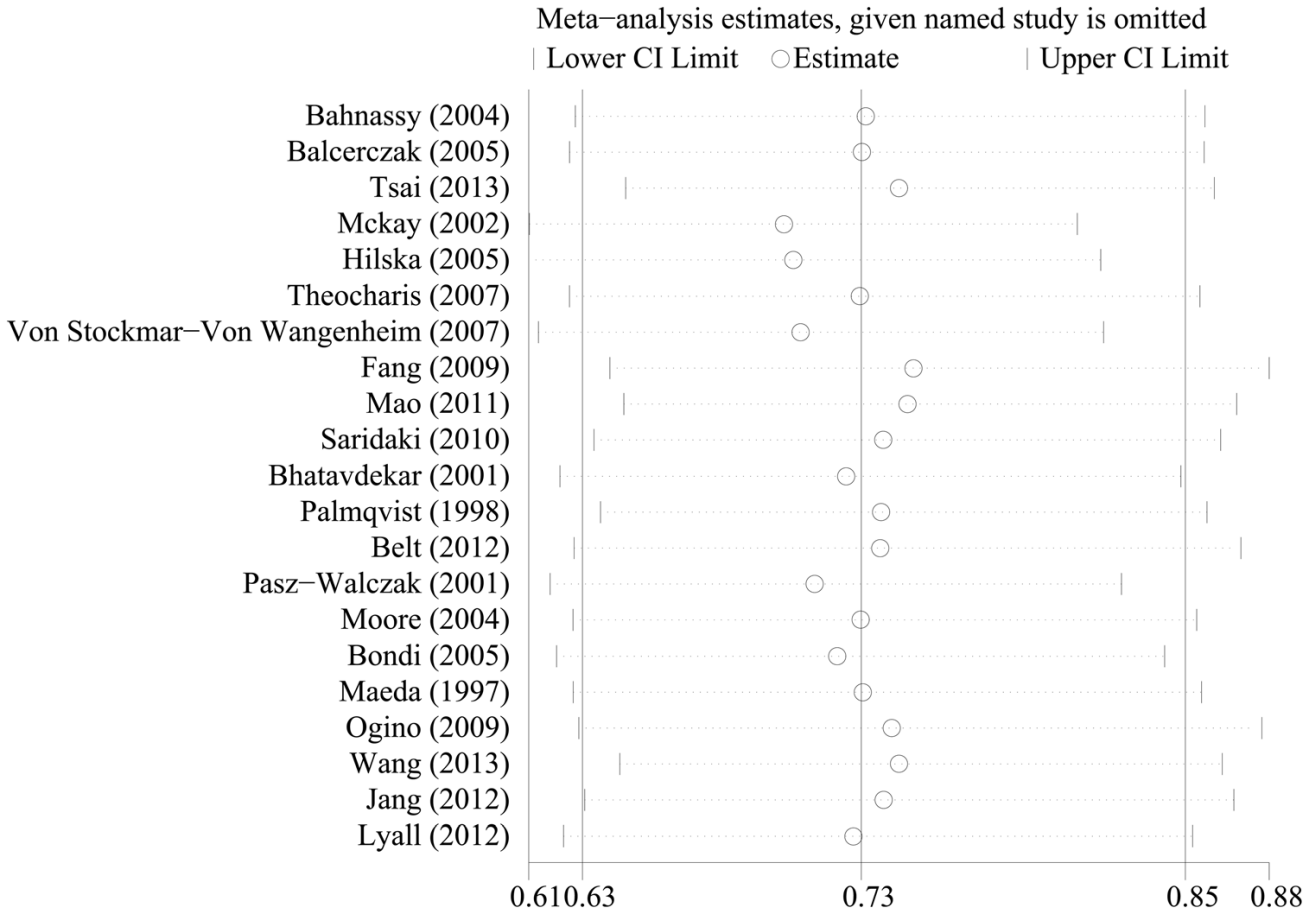
Sensitivity analysis based on stepwise omitting one study at a time for overall survival (OS).

**Figure 5:**
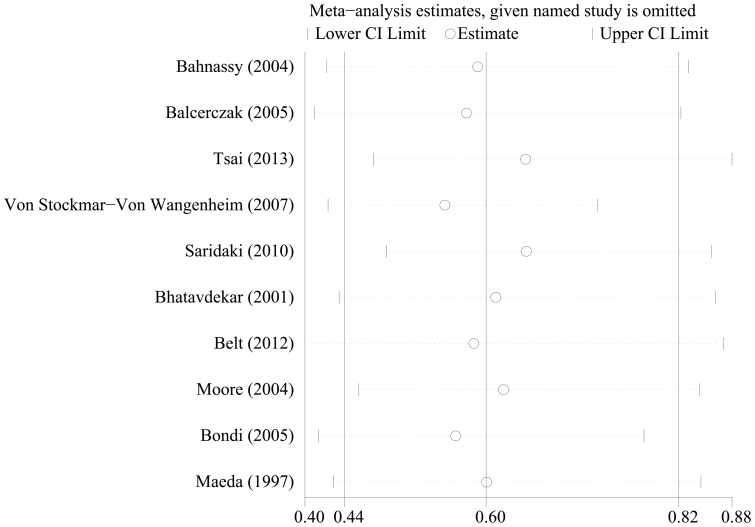
Sensitivity analysis based on stepwise omitting one study at a time for disease-free survival (DFS).

To test the robustness of association between cyclin D1 expression and survival outcome (OS and DFS) and characterize possible sources of statistical heterogeneity, sensitivity analyses were performed by excluding studies one-by-one and analyzing the homogeneity and effect size for all of rest studies. Sensitivity analyses indicated that no significant variation in combined HR by excluding any of the study, confirming the stability of present results (Figure 4 and Figure 5).

### Publication bias

**Figure 6:**
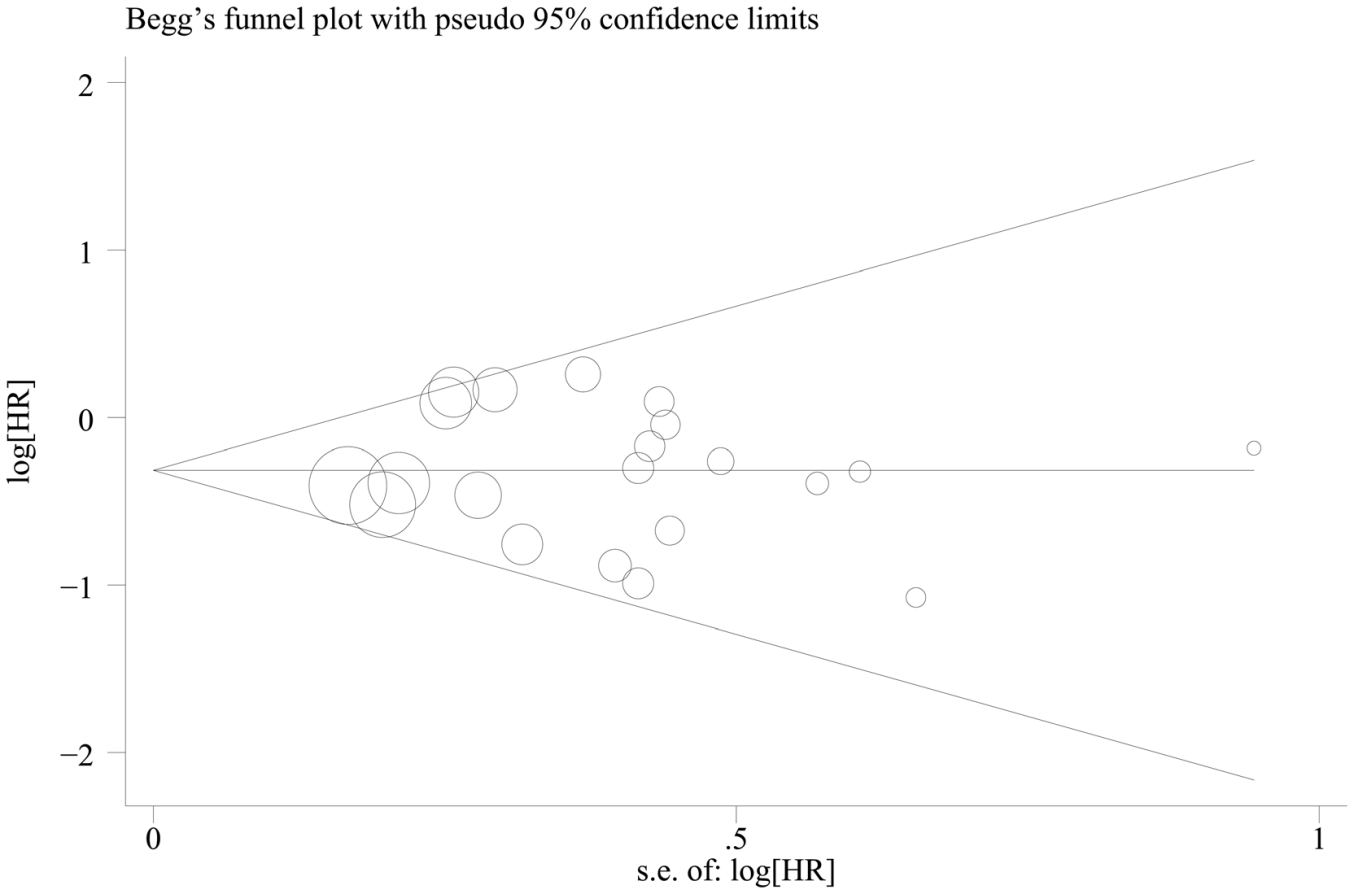
Begg's funnel plot for the evaluation of potential publication bias on overall estimate of overall survival (OS).

**Figure 7:**
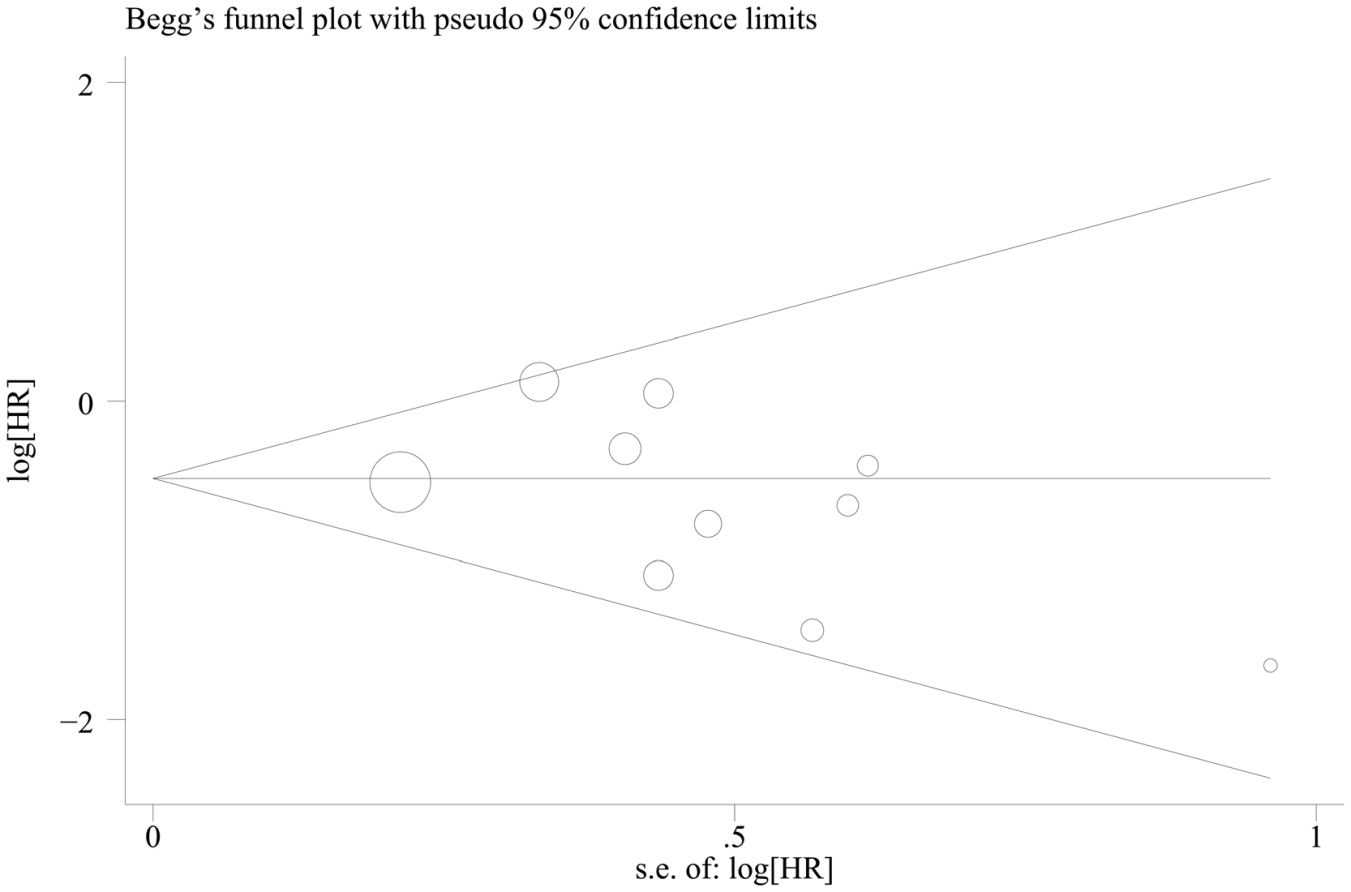
Begg's funnel plot for the evaluation of potential publication bias on overall estimate of disease-free survival (DFS).

Begg's funnel plot and Egger's test were performed to investigate the publication bias of the eligible studies on the summary OS and DFS. The shape of the funnel plot did not reveal any evidence of obvious asymmetry. Then the Egger's test was used to provide statistical evidence of funnel plot symmetry. Twenty one and ten studies investigating cyclin D1 overexpression on OS and DFS yielded an Egger's test score of *P* = 0.886 and *P* = 0.260, respectively, indicating the absence of publication bias in the studies (Figure 6 and Figure 7).

## Discussion

Recently, attention has been drawn at a meta-analytical level on the prognostic marker. Potential roles of cyclin D1 overexpression have been presumed in various types of cancers, including CRC. Zhao *et al*. [36] and Xu *et al*. [37] demonstrated that cyclin D1 overexpression was associated with worst clinicopathological features and prognosis for esophageal squamous cell carcinoma and ER-positive breast cancer. Cyclin D1 overexpression has been reported to occur in 40–70% of colorectal tumors [7], [11], [12], [38]–[40], [47]. Despite a well-established role of cyclin D1 in cell cycle progression, previous data on cyclin D1 and clinical outcome in colorectal cancer have been conflicting. The presence of both significant and non-significant studies addressing the importance of cyclin D1 overexpression in CRC made it necessary to perform a quantitative aggregation of the survival results.

To our knowledge, this is the first meta-analysis on the association between cyclin D1 expression and OS, DFS and the clincopathological parameters in CRC. The present meta-analysis has combined 22 publications including 4150 patients to yield statistics, indicating the cyclin D1 expression is significantly associated with the CRC patients OS and DFS. Subgroup analysis indicated that high cyclin D1 expression was related significantly with poor OS in CRC treated by single surgery and surgery as well as chemoradiation. Cyclin D1 overexpression was also related significantly with poor OS in Asian and non-Asian CRC patients. Besides, high cyclin D1 expression detected by whole tissue sections and tissue microarray was associated with poor OS in CRC patients. Cyclin D1 overexpression based on the nuclear staining was related with a poor OS in CRC patients. In study quality subgroup analysis, both the low quality and high quality studies showed that cyclin D1 overexpresssion had a worse OS. There were also significant relation between cyclin D1-high groups and poor OS in case-control studies and prospective cohort studies. In addition, cyclin D1 overexpression was related significantly with poor DFS not only in patients who received surgery, but also in patients who received surgery and chemoradiation therapies. High cyclin D1 expression was also associated with poor DFS in both Asian and non-Asian patients. High cyclin D1 expression based on nuclear staining was associated with a poor DFS in CRC patients. In study design subgroup analysis, there were significant association between cyclin D1 overexpression and poor DFS in prospective cohort studies but not in case-control studies. A significant relation was also found between cyclin D1 high level and poor DFS in high quality studies but not in low quality studies. Besides, cyclin D1 high expression was related with more older patients (≥60 years), T3,4 category, N positive, distant metastasis patients.

Three patterns of cyclin D1 expression by immunohistochemical method had been found in CRC specimens. Previous studies reported that there existed differences in nuclear cyclin D1 overexpression for colorectal cancer (11–30%) [7], [9], [41]. Cytoplasmic cyclin D1 expression has been shown to be common in non-small lung cancer [42]. Lucas *et al*. suggested that intracellular localization of cyclin D1 is changed during progress through the cell cycle and from the G1-S transition the protein becomes more soluble, reflecting the loss of nuclear cyclin D1 proteins as part reason for cytoplasmic cyclin D1 [43]. Arber *et al*. considered that cytoplasmic localization of cyclin D1 is probably not caused by leakage of protein from the nucleus, since cytoplasmic staining was observed in the total absence of nucleus staining [7]. Bhatavdekar *et al*. showed that cyclin D1 antigen was detected in the cytoplasm of the colorectal cells [13]. Other studies assessed cyclin D1 expression only in the nuclei [9], [14], [16], [18]–[20], [22], [23], [25], [32]–[33], [35], [47]. Nevertheless, some studies demonstrated that cyclin D1 could be detected both in nuclei and cytoplasm in CRC [8], [10], [15], [17], [21], [24], [34]. This present results suggested that only nuclear staining patterns of cyclin D1 overexpression were correlated with the OS and DFS in CRC patients. Thus, large prospective studies taking combinations of three staining patterns to evaluate the OS and DFS in CRC patients into accounts are needed.

Cyclin D1 is the significant prognostic factor for predicting CRC patients' survival. However, co-expression of cyclin D1, p21, PCNA and p53 was previously observed in a subset of patient population [44]; Co-expression of cyclin D1, p21 and PCNA was contributed to the role of cyclin D1 for tumor proliferation, while p53 was inversely associated with cyclin D1 levels, suggesting that overexpression p53 protein is acting to inhibit cellular proliferation [45]. Co-expression of cyclin D1, p21, PCNA and p53, may be an independent prognostic factor for predicting survival. The prognostic value of cyclin D1 in patients with CRC should be examined in the context of other proposed molecular markers such as EGFR, Bcl-2, p21, p53, PCNA, pRb [15], [17], [33], [35]. Only two studies [22], [24] included in the meta-analysis had included a multivariate analysis of co-expression of cyclin D1 and one or several biomarkers. Therefore, large prospective studies taking combinations of cyclin D1 and other most promising markers into account are needed.

Other than displaying cyclin D1 molecule in situ by immunohistochemical staining, some studies have examined cyclin D1 gene or mRNA expression using Southern blot or reverse transcriptase-polymerase chain reaction (RT-PCR) method. Bahnassy *et al*. detected cyclin D1 gene amplification in 50 colorectal cancer cases and found cyclin D1 gene amplification was significantly associated with an advanced disease stage since amplification was detected in 10/15 (66.7%) of stage IV tumors compared to 12/45 (26.7%) of stageI–III tumors. Balcerczak *et al*. used RT-qPCR to quantify cyclin D1 mRNA levels in the investigated colorectal cancers and he found that CCND1 expression was significantly related to lymph nodes and distant metastases. There was also a significant statistical correlation between the presence of CCND1 gene expression and high stages C1, C2, D according to Astler-Cooler's classification [25]. Oda *et al*. assessed cyclin D1 mRNA levels by qRT-PCR in surgically resected specimens of colorectal cancers and observed that the rate of cyclin D1 mRNA expression was significantly higher in patients with venous invasion. Besides, the overexpression of cyclin D1 mRNA was correlated with poor prognosis in CRC patients.

The results should be interpreted cautiously since some limitations exist in this meta-analysis. First, the number of studies and patients classified into the surgery and chemoradiation subgroup of the OS and DFS analysis were limited, respectively. The results upon treatment subgroup analysis should be interpreted with caution. Second, although immunohistochemistry was the most commonly used method for detecting cyclin D1 in situ, RT-qPCR method has also been used for the evaluation of the levels of cyclin D1 gene or mRNA expression in tumor tissue. Studies measuring cyclin D1 gene or mRNA level by RT-qPCR was not yet included in this meta-analysis. Third, another potential source of bias is the variable length of follow-up amongst studies and the differently defined cutoff value. Fourth, the method of obtaining survival data is a potential source of bias. If these statistics were not reported directly by the authors, we calculated from the data available in the article or by extrapolating them from the survival curves, which seemed to be less reliable than when HR was obtained directly from published statistics. These results should be confirmed by well designed prospective studies. Finally, although we did not detect significant heterogeneity or publication bias between studies evaluating the prognostic role of cyclin D1, it is important to note that when the sample size of the studies or the number of primary studies is small, the power to detect potentially important differences is limited. Some important studies had to be excluded from our analysis, for reasons of small size, insufficient survival data, etc. It is known that negative studies are less frequently published or, if they are, with less detailed results, making them less assessable. The missing information reflected “negative” association of cyclin D1 with survival that could decrease the significance of cyclin D1 expression as a predictor of survival outcome. Language bias should not be completed avoided, because of restricted only in English.

In summary, as determined in our meta-analysis, we concluded that cyclin D1 overexpression was significantly associated with poor OS as well as DFS in CRC patients. Cyclin D1 might be an unfavorable prognostic factor for CRC patients. To strengthen our findings, well-designed prospective studies with better standardized assessment of prognostic markers should help to explore the relation between cyclin D1 expression and the CRC patients' outcome.

## Supporting Information

Figure S1 **Forest plot of the odds ratio (OR) for the association of cyclin D1 expression with years of age.**
(TIF)

Figure S2 **Forest plot of the odds ratio (OR) for the association of cyclin D1 expression with tumor size.**
(TIF)

Figure S3 **Forest plot of the odds ratio (OR) for the association of cyclin D1 expression with T category.**
(TIF)

Figure S4 **Forest plot of the odds ratio (OR) for the association of cyclin D1 expression with N category.**
(TIF)

Figure S5 **Forest plot of the odds ratio (OR) for the association of cyclin D1 expression with distant metastasis.**
(TIF)

Figure S6 **Forest plot of the odds ratio (OR) for the association of cyclin D1 expression with histological grade.**
(TIF)

Table S1 **Search strategy in PubMed.**
(DOCX)

Table S2 **Quality assessment of included studies based on the Newcastle-Ottawa Scale.**
(DOCX)

Text S1 **Prognostic significance of cyclin D1 expression in colorectal cancer (Protocol).**
(DOCX)

Checklist S1 **PRISMA checklist.**
(DOC)
