# Supplementary figures and images for: Prognostic Significance of Cyclin D1 Expression in Colorectal Cancer: A Meta-Analysis of Observational Studies
Source: PLoS One. 2014 Apr 11;9(4):e94508. doi: 10.1371/journal.pone.0094508 (PMC3984178; doi:10.1371/journal.pone.0094508)

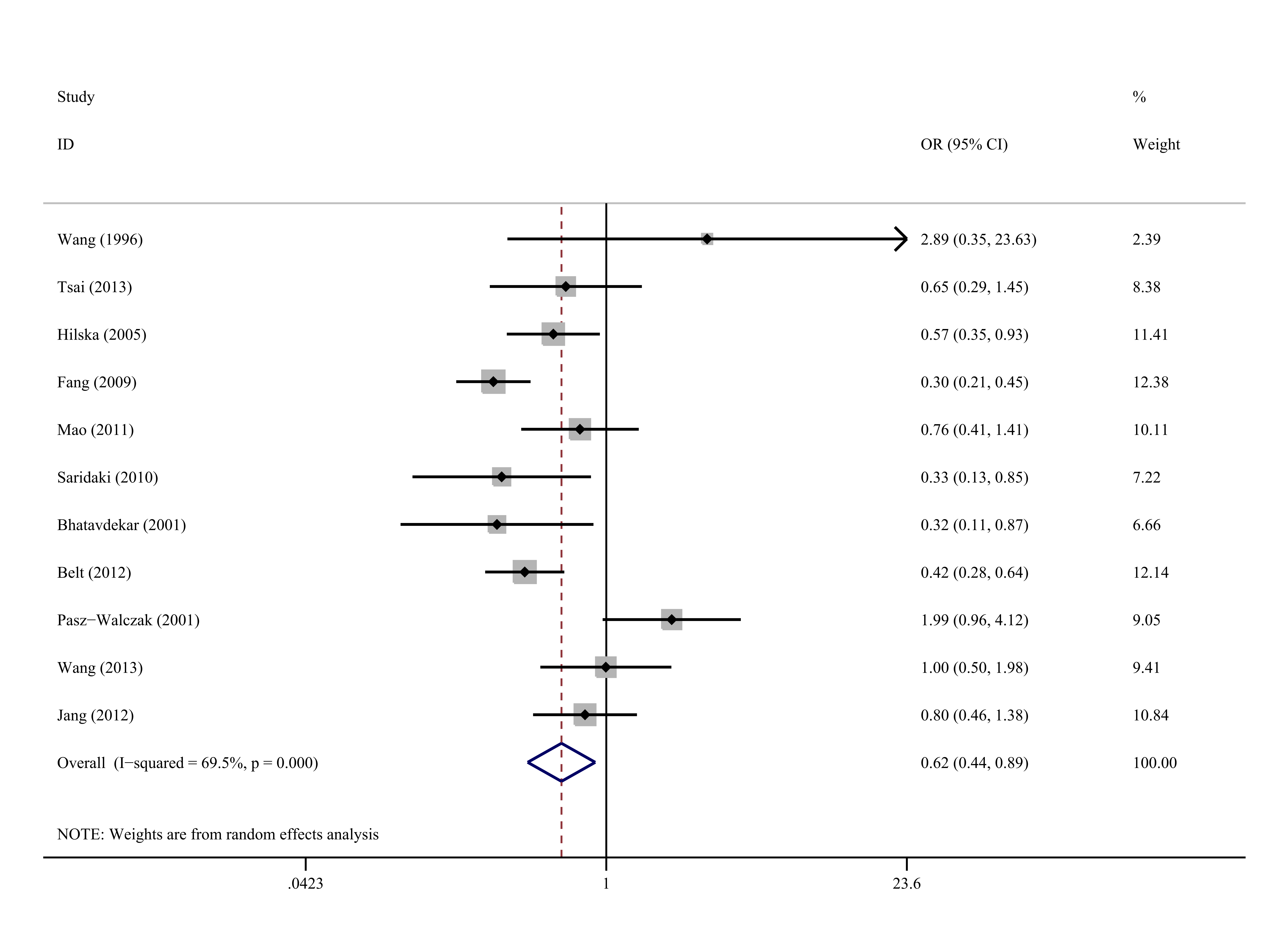

Supplement: Figure S1 — Forest plot of the odds ratio (OR) for the association of cyclin D1 expression with years of age. (TIF) [file pone.0094508.s001.tif]

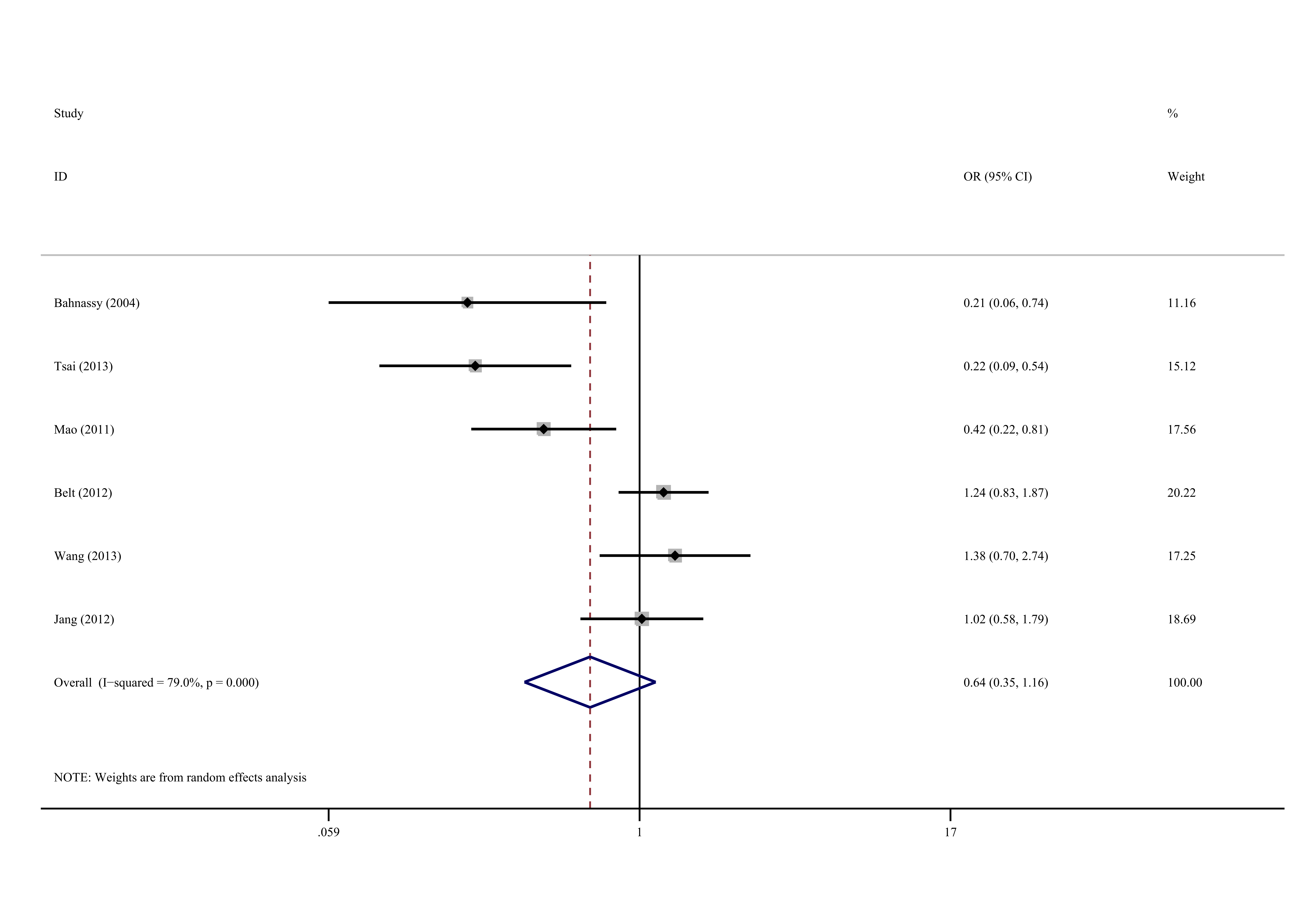

Supplement: Figure S2 — Forest plot of the odds ratio (OR) for the association of cyclin D1 expression with tumor size. (TIF) [file pone.0094508.s002.tif]

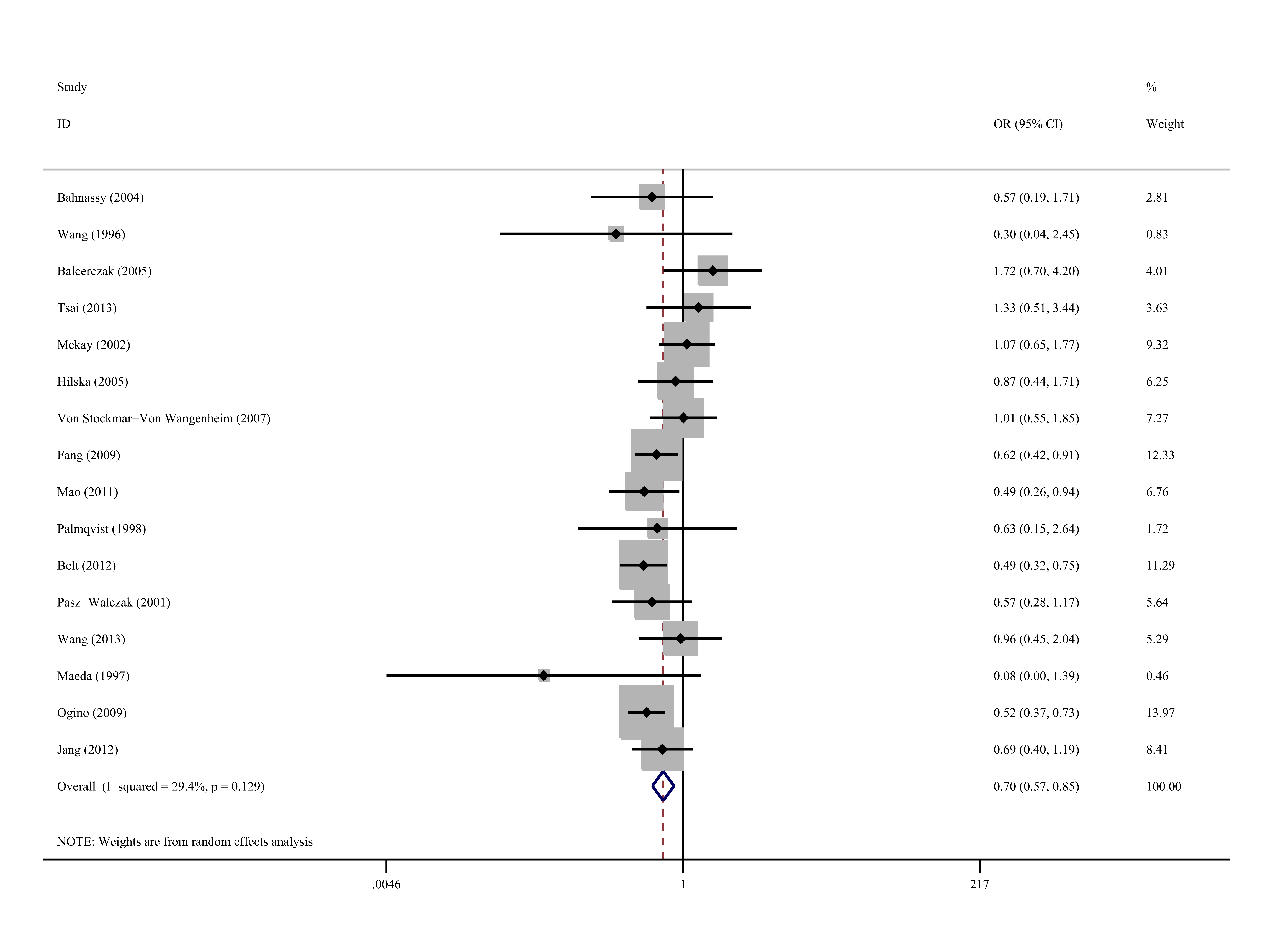

Supplement: Figure S3 — Forest plot of the odds ratio (OR) for the association of cyclin D1 expression with T category. (TIF) [file pone.0094508.s003.tif]

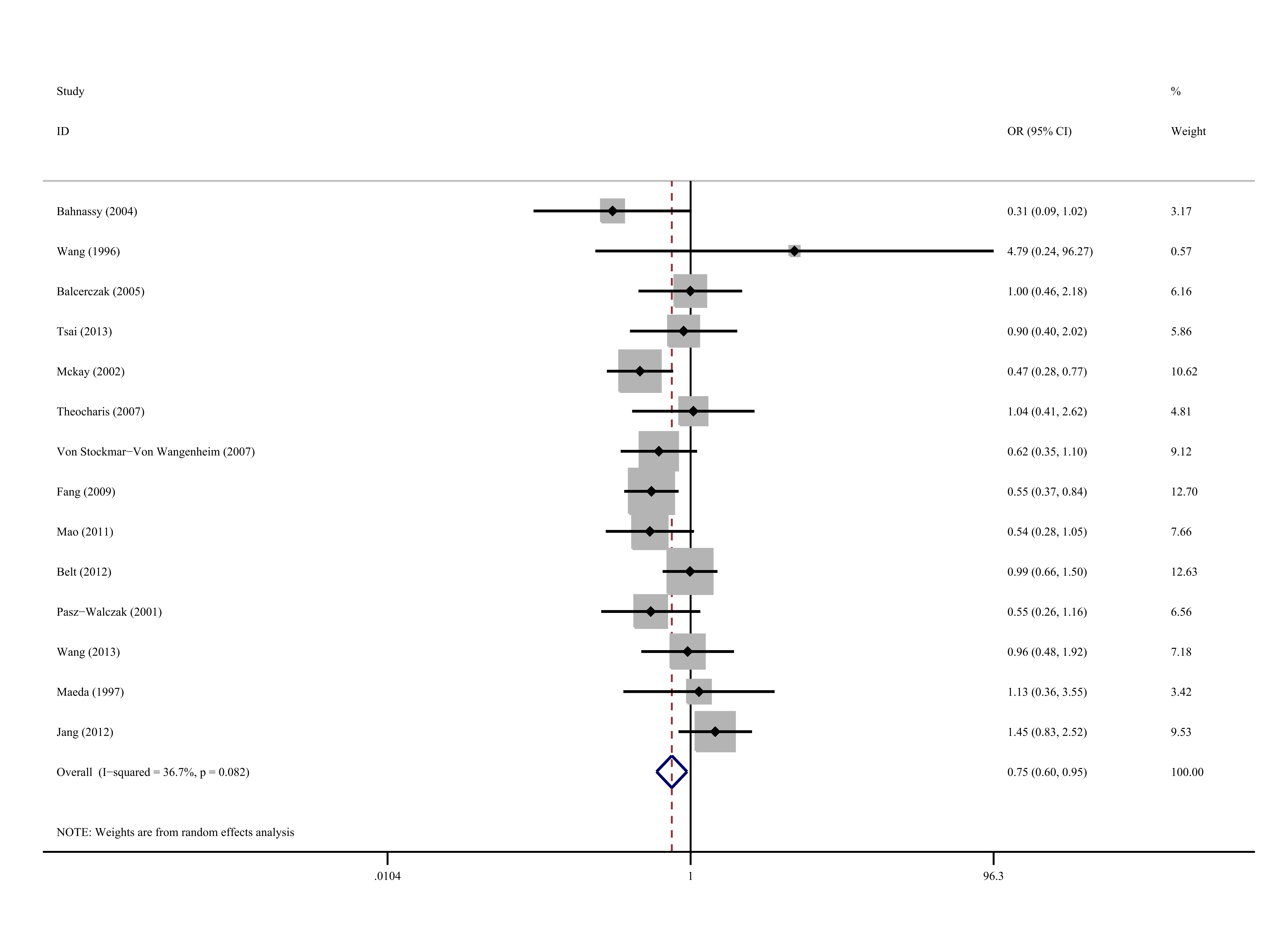

Supplement: Figure S4 — Forest plot of the odds ratio (OR) for the association of cyclin D1 expression with N category. (TIF) [file pone.0094508.s004.tif]

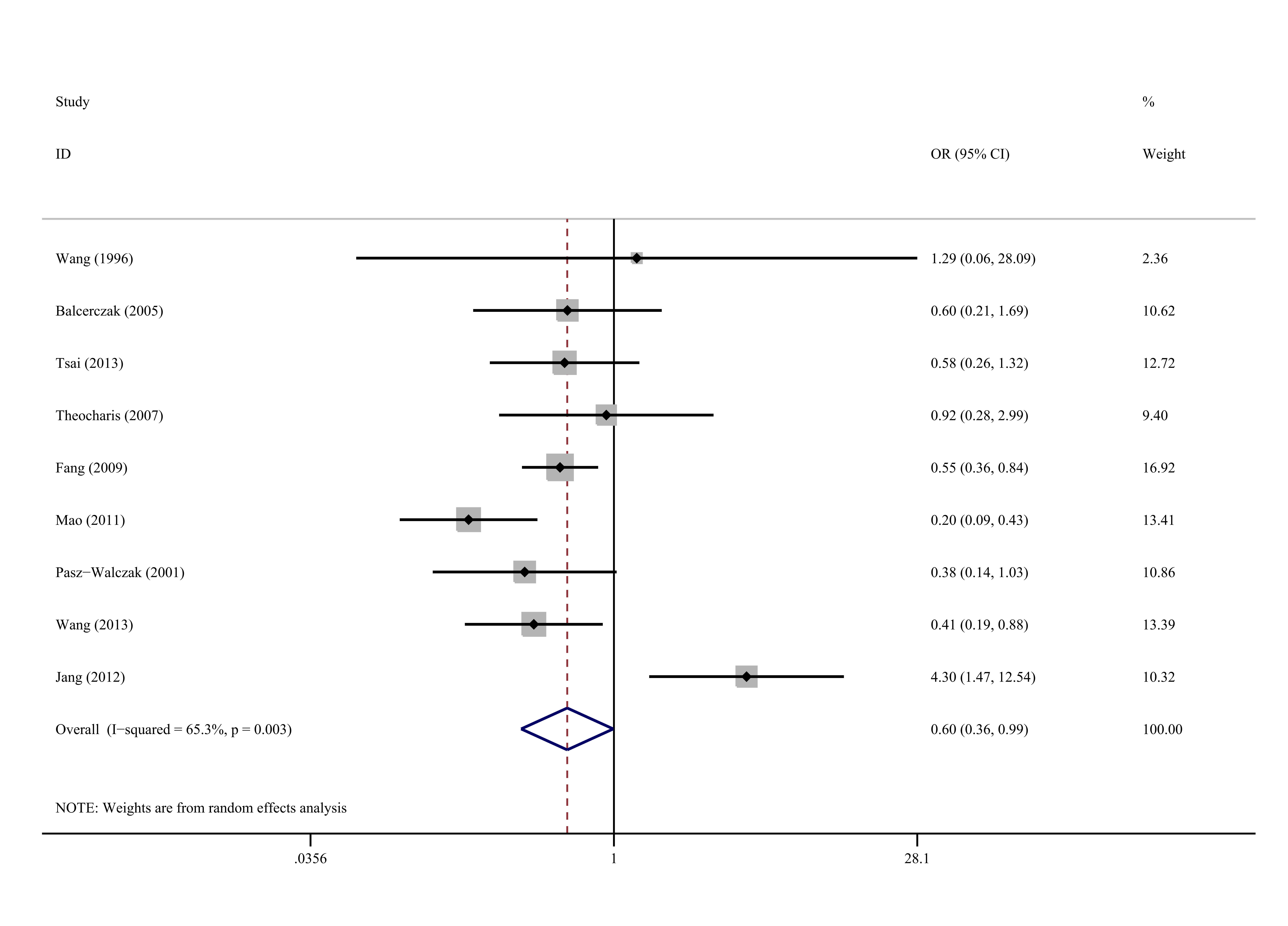

Supplement: Figure S5 — Forest plot of the odds ratio (OR) for the association of cyclin D1 expression with distant metastasis. (TIF) [file pone.0094508.s005.tif]

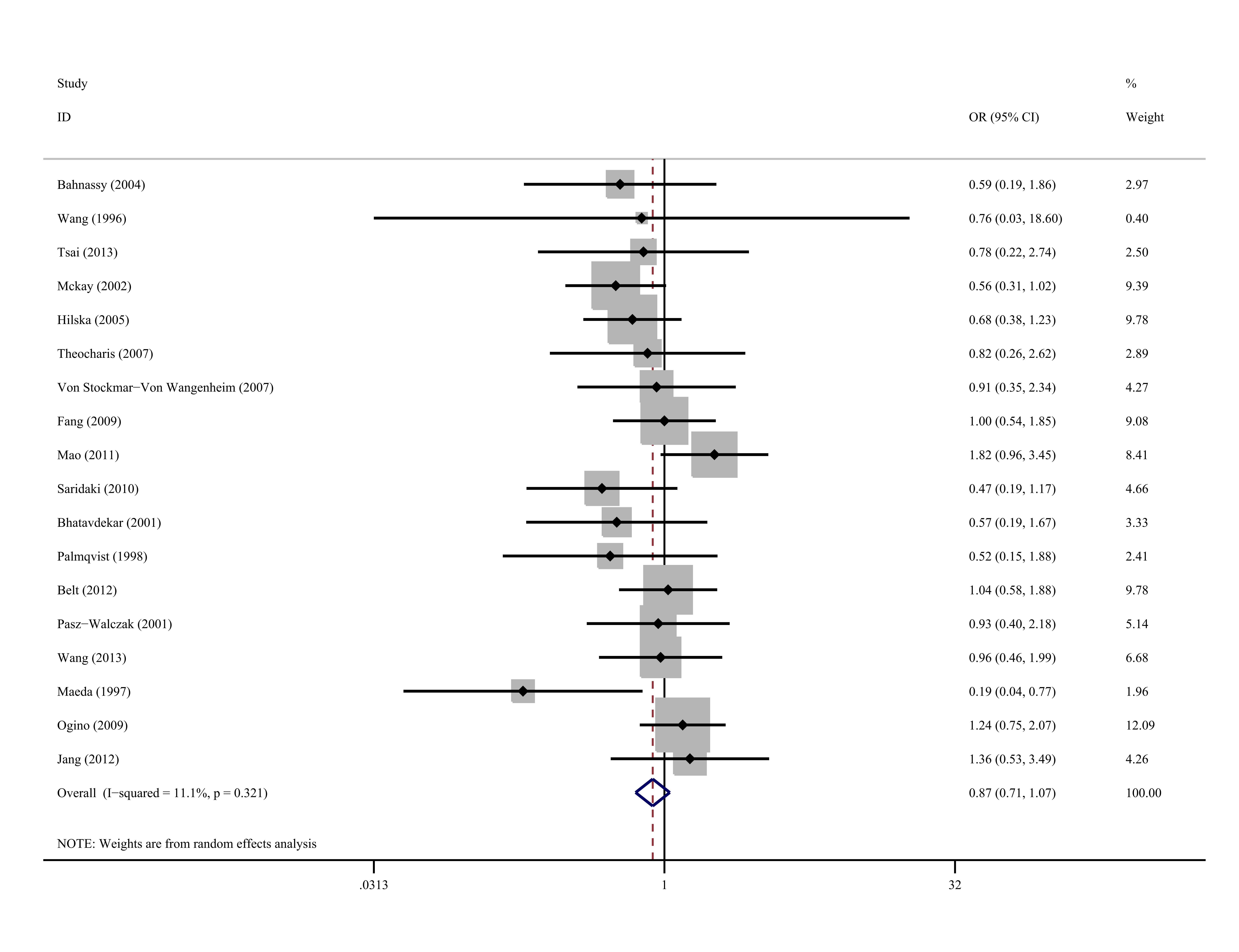

Supplement: Figure S6 — Forest plot of the odds ratio (OR) for the association of cyclin D1 expression with histological grade. (TIF) [file pone.0094508.s006.tif]
