# Supplementary material for: Prognostic Significance of Cyclin D1 Expression in Colorectal Cancer: A Meta-Analysis of Observational Studies
Source: PLoS One. 2014 Apr 11;9(4):e94508. doi: 10.1371/journal.pone.0094508 (PMC3984178; doi:10.1371/journal.pone.0094508)
Supplement: Table S1 — Search strategy in PubMed. (DOCX) [file pone.0094508.s007.docx]

| Table S1. Search strategy in PubMed. | | |
| --- | --- | --- |
| **Search** | **Query** | **Items found** |
|  |  |  |
| #1 | Colorectal neoplasms [MeSH] OR intestinal polyps [MeSH] | 146495 |
|  |  |  |
| #2 | malign* [tiab] OR neoplasm* [tiab] OR carcinoma* [tiab] OR cancer* [tiab] OR tumor* [tiab] OR polyp* [tiab] | 2209597 |
|  |  |  |
| #3 | colon [tiab] OR rectum [tiab] OR rectal [tiab] OR colorectum [tiab] OR colorectal [tiab] OR large bowel [tiab] OR large intestine [tiab] OR gut [tiab] | 303319 |
|  |  |  |
| #4 | #2 AND #3 | 163391 |
|  |  |  |
| #5 | #1 OR #4 | 207566 |
|  |  |  |
| #6 | survival analysis [MeSH] OR survival [MeSH] OR survival rate [MeSH] OR mortality [MeSH] OR recurrence [MeSH] OR neoplasm recurrence [MeSH] OR prognosis [MeSH] OR neoplasm metastasis [MeSH] | 1510741 |
|  |  |  |
| #7 | survival [tiab] OR mortality [tiab] OR recurrence [tiab] OR prognosis [tiab] OR metastasis [tiab] | 1288919 |
|  |  |  |
| #8 | #6 OR #7 | 2263693 |
|  |  |  |
| #9 | CCND 1 protein [MeSH] | 6940 |
|  |  |  |
| #10 | cyclin D1 [tiab] OR cyclin-D1 [tiab] | 11595 |
|  |  |  |
| #11 | #9 OR #10 | 12949 |
|  |  |  |
|  |  |  |
| #12 | #5 AND #8 AND #11 limits: humans | 254 |
| Search time limits: January 2014. | | |
