# Supplementary material for: Prognostic Significance of Cyclin D1 Expression in Colorectal Cancer: A Meta-Analysis of Observational Studies
Source: PLoS One. 2014 Apr 11;9(4):e94508. doi: 10.1371/journal.pone.0094508 (PMC3984178; doi:10.1371/journal.pone.0094508)
Supplement: Table S2 — Quality assessment of included studies based on the Newcastle-Ottawa Scale. (DOCX) [file pone.0094508.s008.docx]

| Table S2. Quality assessment of included studies in the meta-analysis based on the Newcastle-Ottawa Scale. | | | | | | | | | | |
| --- | --- | --- | --- | --- | --- | --- | --- | --- | --- | --- |
| A. Study quality of case-control studies. | | | | | | | | | | |
| **First author** | **Selection** | | | | **Comparability** | | **Exposure** | | | **Total quality scores** |
|  |  |  |  |  |  |  |  |  |  |  |
|  | **Adequate definition of cases** | **Representativeness of cases** | **Selection of controls** | **Definition of controls** | **Study controls for age/gender** | **Study controls for additional factors** | **Exposure ascertainment** | **Same method of ascertainment for cases and controls** | **Non-Response rate** |  |
|  |  |  |  |  |  |  |  |  |  |  |
| Bahnassy, 2004 | ★ | ★ | ★ | ★ | ★ | ★ | — | ★ | — | **7** |
|  |  |  |  |  |  |  |  |  |  |  |
| Wang, 1996 | ★ | ★ | — | ★ | — | — | ★ | ★ | ★ | **6** |
|  |  |  |  |  |  |  |  |  |  |  |
| Balcerczak, 2005 | ★ | — | ★ | ★ | ★ | ★ | ★ | — | ★ | **7** |
|  |  |  |  |  |  |  |  |  |  |  |
| Wang, 2013 | ★ | ★ | ★ | — | — | ★ | ★ | ★ | — | **6** |
|  |  |  |  |  |  |  |  |  |  |  |

| B. Study quality of cohort studies. | | | | | | | | | | |
| --- | --- | --- | --- | --- | --- | --- | --- | --- | --- | --- |
| **First author** | **Selection** | | | | **Comparability** | | **Outcome** | | | **Total quality scores** |
|  |  |  |  |  |  |  |  |  |  |  |
|  | **Representative-ness of the exposed cohort** | **Selection of the non exposed cohort** | **Ascertainment of exposure** | **Demonstration that outcome of interest was not present at start of study** | **Study controls for age/gender** | **Study controls for additional factors** | **Assessment of outcome** | **Adequate duration of follow up (****≥5 years)** | **Adequacy of follow up rate (≥90%) of cohorts** |  |
|  |  |  |  |  |  |  |  |  |  |  |
| Tsai, 2013 | ★ | ★ | ★ | ★ | — | ★ | ★ | — | ★ | **7** |
|  |  |  |  |  |  |  |  |  |  |  |
| Mckay, 2002 | ★ | ★ | ★ | ★ | ★ | ★ | ★ | — | ★ | **8** |
|  |  |  |  |  |  |  |  |  |  |  |
| Hilska, 2005 | ★ | ★ | ★ | ★ | — | ★ | ★ | — | ★ | **7** |
|  |  |  |  |  |  |  |  |  |  |  |
| Theocharis, 2007 | ★ | ★ | — | ★ | ★ | ★ | ★ | — | ★ | **7** |
|  |  |  |  |  |  |  |  |  |  |  |
| Von Wangenheim, 2007 | ★ | ★ | ★ | ★ | ★ | — | ★ | ★ | ★ | **8** |
|  |  |  |  |  |  |  |  |  |  |  |
| Fang, 2009 | ★ | — | ★ | ★ | ★ | — | — | ★ | ★ | **6** |
|  |  |  |  |  |  |  |  |  |  |  |
| Mao, 2011 | ★ | ★ | ★ | ★ | — | ★ | — | ★ | ★ | **7** |
|  |  |  |  |  |  |  |  |  |  |  |
| Saridaki, 2010 | ★ | ★ | ★ | ★ | — | ★ | ★ | — | — | **6** |
|  |  |  |  |  |  |  |  |  |  |  |
|  |  |  |  |  |  |  |  |  |  |  |
|  |  |  |  |  |  |  |  |  |  |  |

| B. Study quality of cohort studies (*Continued*). | | | | | | | | | | |
| --- | --- | --- | --- | --- | --- | --- | --- | --- | --- | --- |
| **First author** | **Selection** | | | | **Comparability** | | **Outcome** | | | **Total quality scores** |
|  |  |  |  |  |  |  |  |  |  |  |
|  |  |  |  |  |  |  |  |  |  |  |
|  | **Representative-ness of the exposed cohort** | **Selection of the non exposed cohort** | **Ascertainment of exposure** | **Demonstration that outcome of interest was not present at start of study** | **Study controls for age/gender** | **Study controls for additional factors** | **Assessment of outcome** | **Adequate duration of follow up (≥5 years)** | **Adequacy of follow up rate (≥90%) of cohorts** |  |
| Bhatavdekar, 2001 | ★ | ★ | — | ★ | — | ★ | ★ | ★ | ★ | **7** |
| Palmqvist, 1998 | ★ | ★ | ★ | ★ | — | ★ | ★ | ★ | — | **7** |
| Belt, 2012 | ★ | ★ | ★ | ★ | ★ | ★ | ★ | — | ★ | **8** |
| Pasz-Walczak, 2001 | ★ | ★ | ★ | ★ | — | ★ | ★ | — | ★ | **7** |
|  |  |  |  |  |  |  |  |  |  |  |
| Moore, 2004 | ★ | ★ | ★ | ★ | — | ★ | ★ | ★ | ★ | **8** |
|  |  |  |  |  |  |  |  |  |  |  |
| Bondi, 2005 | ★ | ★ | ★ | — | ★ | — | ★ | ★ | — | **6** |
|  |  |  |  |  |  |  |  |  |  |  |
| Jang, 2012 | ★ | ★ | ★ | — | ★ | ★ | ★ | — | ★ | **7** |
|  |  |  |  |  |  |  |  |  |  |  |
| Lyall, 2012 | ★ | ★ | — | ★ | ★ | ★ | ★ | ★ | ★ | **8** |
|  |  |  |  |  |  |  |  |  |  |  |
| Maeda, 1997 | ★ | ★ | ★ | ★ | — | ★ | ★ | ★ | — | **7** |
|  |  |  |  |  |  |  |  |  |  |  |
| Ogino, 2009 | ★ | ★ | — | ★ | ★ | ★ | ★ | ★ | ★ | **8** |
|  |  |  |  |  |  |  |  |  |  |  |
