# Supplementary material for: Prognostic Significance of Cyclin D1 Expression in Colorectal Cancer: A Meta-Analysis of Observational Studies
Source: PLoS One. 2014 Apr 11;9(4):e94508. doi: 10.1371/journal.pone.0094508 (PMC3984178; doi:10.1371/journal.pone.0094508)
Supplement: Text S1 — Prognostic significance of cyclin D1 expression in colorectal cancer (Protocol). (DOCX) [file pone.0094508.s009.docx]

## **Prognostic significance of cyclin D1 expression in colorectal cancer: A meta-analysis (Protocol)**

**Objectives:**

Cyclin D1 is a member of cyclins. It has been considered to be an oncogene which could regulate progression from the G1 phase of the cell cycle to the S phase. Cyclin D1 expression is known to be upregulated in a variety of tumor types and cyclin D1 overexpression occurs in one-third or more of colorectal cancers. Many retrospective studies have evaluated whether cyclin D1 overexpression may be a prognostic factor for survival in patients with colorectal cancer (CRC). However, the results of the studies are inconclusive and no consensus has been reached. Bahnassy *et al*. and Maeda *et al*. reported that cyclin D1 overexpression has been associated with poor prognosis, while Mckay *et al*. draw a conclusion that the high level of cyclin D1 indicate good prognosis. A few studies have shown no prognostic value of cyclin D1 overexpression in CRC patients.

The objective of this meta-analysis is to evaluate the association between the expression of cyclin D1 and the outcome of CRC patients and clinicopathological features.

**Methods: Criteria for considering studies for this meta-analysis**

**Eligibility criteria for study:** Peer reviewed articles or abstracts of observational studies such as cohort studies and case-control studies to evaluate cyclin D1 expression in colorectal cancer with immunohistochemistry (IHC) or tissue microarray. The endpoint of studies investigated was OS or DFS. The study should also report a hazard ratio (HR) and 95% confidence interval (CI) or the data sufficient to estimate the HR and 95% CI from survival analysis.

**Eligibility criteria for the patient population:** Patients had received complete surgical resection of the tumor, which was pathologically diagnosed CRC.

**Eligibility criteria for each intervention and comparator:** Cyclin D1-high group could be defined detection of cyclin D1 expression using immunohistochemistry or tissue microarray in nuclear, cytoplasmic or both of them. The method of defining cyclin D1-low group was identical to cyclin D1-high group. Since the cutoff value for cyclin D1-high group varied with different studies, we defined cyclin D1-high expression values according to the original articles.

**Methods: Search methods for identification of studies:**

**Databases:** PubMed, EMBASE and ISI Web of science were searched to identify studies assessing cyclin D1 as prognostic factor in patients with colorectal cancer. The upper data limit of January 2014 was applied, with no lower data limit.

**Key words:** “cyclin D1”, “CCND 1”, “prognosis”, “colorectal cancer”

Reference lists of original articles and review articles were also examined for additional literature.

Dr. Yang Li, Dr. Jun Wei independently searched and screened the eligible studies and abstracted the data. Dr. Yang Li and Dr. Chuanhui Xu analyzed the data. Professor Zhongxin Zhao and Professor Tiangeng You reviewed the process to ensure the eligibility of the studies.

**Methods: Data collection and analysis:**

| Final list of eligible studies. | | | | |
| --- | --- | --- | --- | --- |
| **First author/year** | **Country** | **Number of patients** | **Location of cancer** | **Duration of follow-up** |
|  |  |  |  |  |
| Bahnassy/2004 | Egypt | 60 | Colorectal | * |
|  |  |  |  |  |
| Wang/1996 | Japan | 39 | Colorectal | * |
|  |  |  |  |  |
| Balcerczak/2005 | Poland | 111 | Colorectal | * |
|  |  |  |  |  |
| Tsai/2013 | Taiwan, China | 100 | Colorectal | Median 30.5 months |
|  |  |  |  |  |
| Mckay/2002 | UK | 249 | Colorectal | Median 35 months |
|  |  |  |  |  |
| Hilska/2005 | Finland | 363 | Colorectal | NA |
|  |  |  |  |  |
| Final list of eligible studies (*Continued*). | | | | |
| **First author/year** | **Country** | **Number of patients** | **Location of cancer** | **Duration of follow-up** |
| Theocharis/2007 | Greece | 86 | Colon | Median 43 months |
|  |  |  |  |  |
| Von Wangenheim/2007 | Germany | 200 | Colorectal | At least 5 years |
|  |  |  |  |  |
| Fang/2009 | China | 532 | Colon | Median 52 months |
|  |  |  |  |  |
| Mao/2011 | China | 169 | Colon | 3 to 107 months |
|  |  |  |  |  |
| Saridaki/2010 | Greece | 144 | Colorectal | NA |
|  |  |  |  |  |
| Bhatavdekar/2001 | India | 98 | Colorectal | 5 years |
|  |  |  |  |  |
| Palmqvist/1998 | Sweden | 90 | Colorectal | Median 42 months |
|  |  |  |  |  |
| Belt/2012 | Netherland | 379 | Colon | NA |
|  |  |  |  |  |
| Pasz-Walczak/2001 | Poland | 122 | Colorectal | Median 44.5 months |
|  |  |  |  |  |
| Moore/2004 | US | 40 | Rectum | Median 69 months |
|  |  |  |  |  |
| Bondi/2005 | Norway | 219 | Colon | 5 years |
|  |  |  |  |  |
| Maeda/1997 | Japan | 101 | Colorectal | 5 years |
|  |  |  |  |  |
| Ogino/2009 | US | 602 | Colon | Every 2 years |
|  |  |  |  |  |
| Wang/2013 | China | 139 | Colorectal | * |
|  |  |  |  |  |
| Jang/2012 | Korea | 217 | Colorectal | NA |
|  |  |  |  |  |
| Lyall/2012 | United Kingdom | 90 | Colorectal | 60 to 100 months |
|  |  |  |  |  |
| * These studies looked back at medical records and did not report the time of follow-up.  NA, not available. | | | | |

**Data collection:** Dr. Yang Li and Dr. Jun Wei will collect the data using the predefined data collection form. Disagreements were resolved with third author (Tiangeng You) by discussion. When the data we need are not available from the published paper, Yang Li is responsible for contacting the original researchers to get their help.

**Extracted data:** The first author, year of publication, study location, number of participants, staining patterns of cyclin D1, the choice of cutoff scores for the definition of positive staining or staining intensity, antibody used, antibody working concentration, duration of follow-up, T category, N category, distant metastasis, histology, and prognostic outcomes of interest (DFS and/or OS).

**Predefined subgroups for OS and DFS:** Treatment (surgery versus surgery and chemoradiation), ethnic (Asian versus non-Asian), Samples (whole tissue sections versus tissue microarray), immunohistochemical staining patterns (nuclear versus cytoplasmic versus both of them), quality scores of included studies (≥ 7 versus < 7), study design (cohort study versus case-control study).

**Analysis:** The OS and DFS will be estimated by collection of HR with 95% confidence interval (CI) which were presented in the original publications or obtained from available data using methods reported by Tierney *et al*. Kaplan-Meier curves were read using Engauge Digitizer version 4.1 (<http://digitizer.sourceforge.net/>).

The quality of included studies was assessed based on a 9-scores system of the Newcastle-Ottawa Scale (NOS). Each study included in the meta-analysis was judged on three broad perspectives: (1) the selection of the groups of study (four items, one score each), (2) the comparability (one item, up to two scores) and (3) the ascertainment of either the exposure or outcome of interest (three items, one score each). A score presents a high quality choice of individual study. The total number of stars was accumulated, with more stars reflecting a higher methodological quality. A study could be awarded a maximum of nine stars. In this 9-scores system, studies scored equal or greater than 7 were considered as high quality.

The individual HR estimates were pooled into a summary HR by using DerSimonian and Laird random-effects methods. The random-effects model, which not only weights each study by its inverse variance but also includes the within- and between-studies variances and usually is more conservative, was chosen. Statistical heterogeneity assessment between studies was performed by using a chi-square heterogeneity statistic based *Q* test. Given the low test power, the significance level was defined as *P* < 0.10. The effect of heterogeneity was also quantified using the inconsistency index (*I*^2^). The *I*^2^ statistic is defined as the percentage of total variance across studies attributable to heterogeneity rather than the chance [*I*^2^ = (*Q* – df)/*Q* × 100%]. As a guide, *I*^2^ values of < 25% may be considered “low”, values of 25-50% may be considered “moderate” and values of > 50% may be considered “high”. The potential for publication bias was assessed by using the Begg rank correlation method and the Egger weighted regression method. Sensitivity analysis was performed to evaluate the influence of a single study on the overall effect estimate by excluding one study at a time. The statistical tests mentioned above were performed by STATA version 11.0 (Stata Corporation, College Station, TX).
